# Targeting high endothelial venules: potential strategies for cancer treatment

**DOI:** 10.1080/07853890.2025.2597587

**Published:** 2025-12-02

**Authors:** Weichang Yang, Guofeng Zhu, Shanshan Cai, Hongquan Xing, Aoyu Yang, Xiaoqun Ye

**Affiliations:** Department of Respiratory and Critical Care Medicine, The Second Affiliated Hospital of Nanchang University, JiangxiMedical College, Nanchang University, Nanchang, China

**Keywords:** High endothelial venule, lymph node metastasis, treatment, cancer

## Abstract

**Background:**

High endothelial venules (HEVs) are specialized blood vessels found primarily in lymphoid tissues, notably at the cortico-paracortical junction of lymph nodes (LNs). They play a key role in lymphocyte trafficking, facilitating the entry of lymphocytes into peripheral lymphoid organs.

**Objective:**

Recent evidence indicates the presence of HEVs within tumor tissues, where they form part of tertiary lymphoid structures. This review aims to explore the structural and functional alterations of HEVs in tumors and LNs, and investigate their role in cancer prognosis and response to immunotherapy.

**Methods:**

We conducted a comprehensive review of recent studies examining HEVs in tumors and LNs, with a focus on their involvement in immune responses and cancer progression. Additionally, the use of MECA-79 as a specific HEV marker was considered as a potential therapeutic target.

**Results:**

HEVs within tumors have been associated with improved immune surveillance and better prognosis in certain cancers. The presence of HEVs in tumors correlates with enhanced immune cell infiltration and may influence the effectiveness of immunotherapy treatments. Targeting HEVs, particularly through MECA-79, holds promise as a novel therapeutic strategy to modulate tumor immunity.

**Conclusion:**

Targeting HEVs in tumor tissues represents a promising avenue for cancer therapy. Further research is needed to optimize strategies for modulating HEV function and to better understand their role in immune responses to cancer.

## Introduction

High endothelial venules (HEVs) are highly specialized vascular structures composed of high endothelial cells (HECs). The shape of HECs was first described as plump and columnar, with significant morphological differences compared to other vascular endothelial cells [1]. HEV endothelial cells are termed ‘high’ due to their distinctive tall, cuboidal morphology, which is essential for their specialized function in lymphocyte trafficking. This phenotype is driven by specific signaling pathways, including interactions between endothelial cells and surrounding stromal cells, as well as the action of transcription factors like NF-κB. Mionnet et al. describe how LTβR signaling and NF-κB activation are involved in the morphological differentiation of these endothelial cells, allowing them to facilitate the selective entry of lymphocytes into lymphoid tissues [2]. It is well known that lymphoid tissues, as the major immune organs in the body, primarily exert immune responses through immune cells in the tissue, such as dendritic cells (DCs), T cells, and B cells [3]. HEVs are unique small venules mainly found in lymph nodes (LNs) and secondary lymphoid organs. They are located in the III-IV level structures of the LN vascular system and are responsible for guiding lymphocytes to migrate from the bloodstream into lymphoid tissues, a key mechanism of HEV-mediated lymphocyte homing [4].

Due to the distinct function of HEVs in LNs, when their structure or function changes, it may lead to a decrease in the lymphocyte content that home to the LNs, thereby exhausting the immune function of the LNs and creating favorable conditions for tumor cells [5]. In recent years, studies have shown that HEVs not only exist in lymphoid tissues and secondary lymphoid organs but also appear in tertiary lymphoid structures (TLS) within tumors. They were first reported in solid tumors and are considered tumor-associated HEVs (TA-HEVs) [6]. Unlike HEVs in LNs, the development of HEVs within tumors may increase the recruitment of lymphocytes, which could be beneficial, as HEVs may mediate the recruitment of lymphocytes within tumors, enhancing anti-tumor effects [7].

Because of their unique role in mediating lymphocyte homing, HEVs play different roles in LNs and primary tumors as part of TLS. This suggests that HEVs could be an effective target for preventing tumor metastasis and treating tumors. Based on the morphological and functional changes of HEVs in LNs and tumor tissues [8], this article discusses the potential role of HEVs in cancer treatment and prevention of LN metastasis.

## Localization and structure of HEVs

HEVs are primarily distributed in secondary lymphoid organs, such as LNs and tonsils. In these locations, HEVs are responsible for guiding lymphocytes from the bloodstream into lymphoid tissues, thereby facilitating immune surveillance and immune responses. HEVs are mainly found in the cortex of LNs, particularly in the T cell area [9]. HEVs are specialized blood vessels found primarily in secondary lymphoid organs (SLOs) such as LNs, Peyer’s patches. They are not present in all lymphoid tissues, such as the thymus and bone marrow, which are considered primary lymphoid organs [10]. In LNs, HEVs are situated at the interface between the cortex and paracortex as well as within the paracortical region, where T cells reside. They form part of the specialized venular network, which consists of a hierarchical system of postcapillary venules that branch from the largest collecting venules (grade I) down to the smallest postcapillary segments [11]. The columnar endothelial structure of HECs facilitates the migration of lymphocytes across intercellular gaps, allowing lymphocytes to pass through the endothelial cells with minimal vascular leakage [12]. Direct activation of LTβR on HECs is essential for the development of cuboidal HEVs and for establishing their lymphocyte-trafficking properties, including polarized ICAM1 expression, CCL21 production, and the formation of lymphocyte-retaining pockets [13].

The most distinctive feature of HECs is their high columnar endothelial structure, which distinguishes them from other vascular endothelial cells. At the ultrastructural level, HECs exhibit characteristics of metabolically active, secretory cells, with prominent Golgi apparatus, abundant mitochondria, rough endoplasmic reticulum, and clusters of ribosomes, along with large, round nuclei [14]. Recent studies have shown that tumor-associated blood vessels displaying the ‘MECA-79’ marker, similar to those found in HEVs, have a phenotype different from another tumor vasculature [15]. Researchers have found that this type of tumor vasculature is significant because it helps inhibit tumor growth rather than promoting it.

## Specific markers of HEVs

The interaction between naive lymphocytes and HEVs is initiated by L-selectin (CD62L), which can recognize the sulfated mucin-like glycoprotein family of HEV sialomucins [16]. CD62L is highly expressed on naïve T cells and is essential for their homing to LNs *via* interactions with the PNAd glycoproteins on HEVs. In contrast, effector T cells downregulate CD62L expression, which limits their ability to enter LNs and instead promotes trafficking to peripheral tissues, where they perform immune effector functions [17]. This regulation of CD62L expression is crucial for understanding the dynamics of T cell homing and the functional significance of HEV development, particularly in tumors. In the tumor microenvironment, the presence or absence of HEVs can influence T cell infiltration and, consequently, the efficacy of immune responses. This interaction is a critical step in the process of lymphocyte homing. Specific markers of HEVs include CD34, Chst4, Fut7, Nepmucin, and Glycam1, which, when translated and modified by enzymes highly expressed in HECs, become effective ligands for CD62L [18]. CD34 is widely expressed on the surface of vascular endothelial cells and hematopoietic progenitor cells [19]. However, it only acts as a ligand for CD62L after being specifically sulfated, fucosylated, and sialylated by HEC enzymes.

MECA-79 antibodies are currently the most widely used markers for identifying HEVs *in vivo* [20]. These antibodies were generated and first reported by Butcher et al. in 1988. PNAd (Peripheral Node Addressin) refers to a group of glycoproteins expressed on HEVs in peripheral lymph nodes. These glycoproteins share a common carbohydrate epitope, which is recognized by the MECA-79 monoclonal antibody. The sialyl Lewis-X epitope, important for CD62L binding, is a crucial component of this carbohydrate structure, facilitating leukocyte trafficking to lymphoid tissues [21]. However, MECA-79 is not only used to identify HEVs in LNs but also reacts with HEV-like vasculature in other lymphoid tissues. Therefore, while PNAd is widely used for detecting MECA-79-reactive antigens in lymphoid and non-lymphoid organs, MECA-79 may be a more suitable marker for defining HEVs [22]. This is because it is a very stable monoclonal antibody that can be used in various detection methods and specifically reacts with HEVs (without cross-reacting with other blood vessels) in both humans and mice, as well as with both lymphoid and non-lymphoid organs [23].

## Regulatory mechanisms of HEV homeostasis

Previous studies have shown that ligation of the afferent lymphatic vessels in LNs leads to the dedifferentiation of HEVs [24]. This phenomenon is characterized by a ‘flattening’ of HEV morphology, downregulation of MECA-79 antigen, and a decrease in lymphocyte homing function [25]. However, HEV function can be fully restored after the recovery of the lymphatic vessels. Further research revealed that chemokines can reach HEVs through a stromal conduit system composed of fibroblasts in the LN, highlighting the special relationship between HEVs and the lymph from draining tissues [26]. Phenotypic analysis of HEVs indicated that freshly purified human HECs rapidly lose their specialized columnar phenotype in *ex vivo* culture [27], suggesting that HEVs exhibit significant plasticity, primarily in their morphological features, and are highly dependent on LN cells and chemokines for maintenance.

It has been found that DCs are essential for maintaining the normal morphology and function of HEVs in steady-state LNs [28]. In fact, the depletion of CD11c^+^ DCs in LNs induces the differentiation of HEVs into an immature phenotype, characterized by decreased MECA-79 antigen expression, downregulation of HEV-specific genes (such as Chst4, Fut7, and Glycam1), and upregulation of Madcam1, which has previously been reported as a marker for immature HEVs [29]. The differentiation of steady-state HEVs into an immature phenotype is considered a process of HEV dedifferentiation, leading to a significant impairment of lymphocyte homing to LNs and eventually resulting in a decrease in lymphocytes in the LNs. Further studies have shown that DCs promote HEV growth in a VEGF-dependent manner, indicating that DCs have an additional regulatory role in HEV function [30].

**Figure 1.**
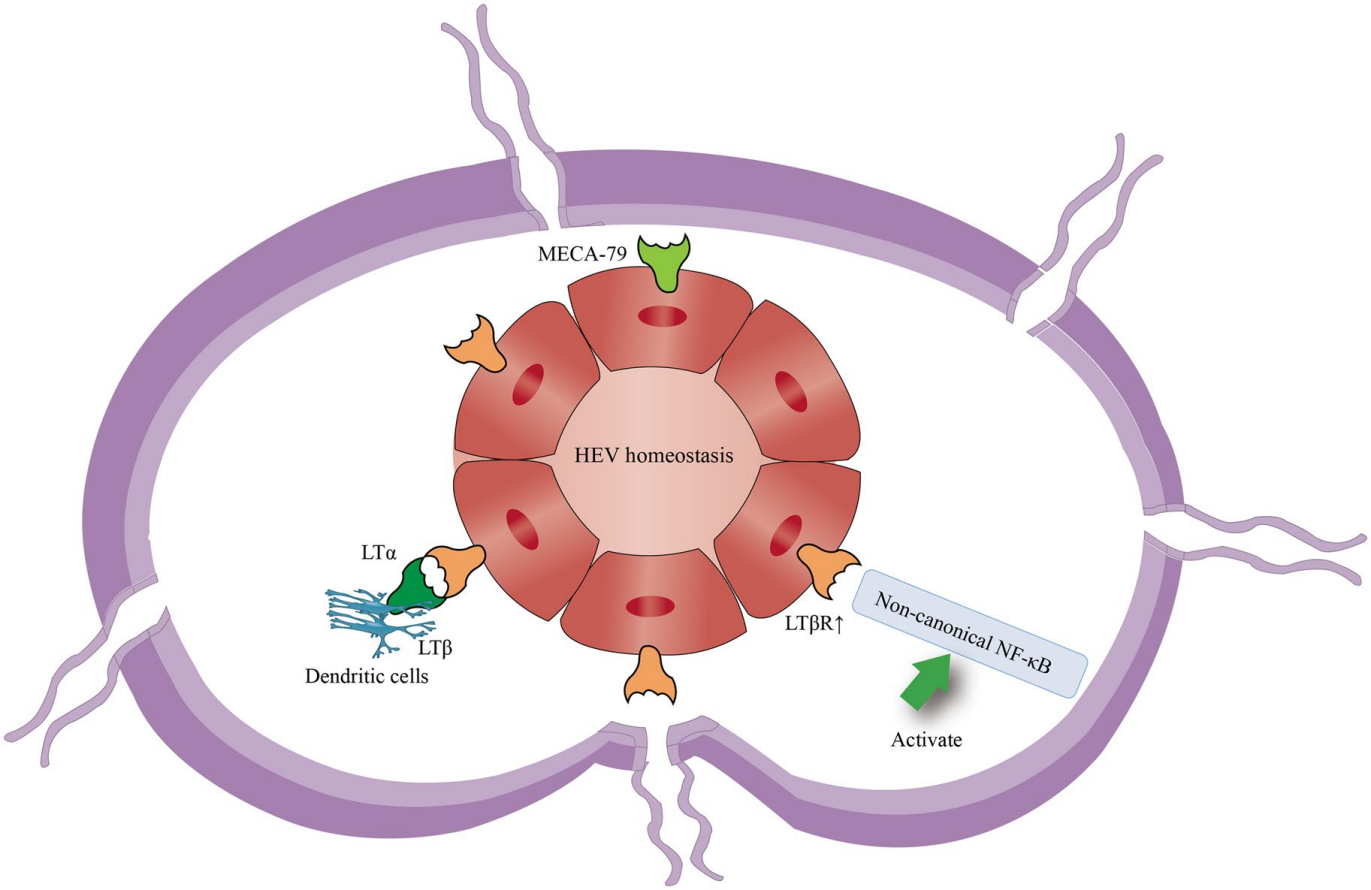
Regulatory mechanisms of HEV homeostasis.

The LTβR and NF-κB signaling pathways are well-established as crucial for the maintenance of HEVs and lymphocyte homing to LNs [31]. However, the roles of LTβR and TNFR1 in HEVs remain a subject of ongoing debate and investigation. Studies involving endothelial cell-specific knockout of LTβR and soluble LTβR-Ig decoy receptor treatment in mice showed that continuous activation of LTβR on the surface of HECs is essential for maintaining HEV morphological features and the expression of biologically relevant markers (such as Glycam1, Fut7, Chst4, and Gcnt1) [13], suggesting that LTβR is a key target for maintaining the high columnar endothelial characteristics of HEVs, lymphocyte homing, and HEV markers [32]. However, in TNFR1 knockout mice and mice treated with TNFR-Ig, the HEV phenotype and expression of HEV-specific genes were unaffected. In contrast, mice with defects in the non-canonical NF-κB signaling pathway showed reduced expression of MECA-79 antigen, Glycam1, and Chst4 in LN HEVs, indicating that the ability of LTβR to induce the non-canonical NF-κB signaling pathway is crucial for HEV homeostasis regulation (Figure 1). Notably, researchers have found that CD11c^+^ DCs are the primary source of LTβR ligands [28], including LTα, LTβ, and LIGHT, and that DC-derived lymphotoxins are critical for HEV-mediated lymphocyte recruitment to steady-state LNs [33].

## HEVs in tumor draining lymph nodes

Tumor-draining lymph nodes (TDLNs) are defined as the first station where tumor cells metastasize to LNs, acting as a key transit point for tumor cell spread to distant organs. Their function is double-edged [34]. TDLNs are not only the site where lymphocytes initiate immune responses against tumors but also serve as a transfer point for tumor cells to spread into the bloodstream. Most solid tumor cells enter the TDLN through lymphatic vessels before spreading to distant organs, forming LN metastases [35]. Subsequently, tumor cells exit the TDLN through efferent lymphatic vessels and enter the bloodstream *via* the thoracic duct, further disseminating to distant organs. Overall, the anatomical location of the TDLN determines its potential value in cancer therapy [36].

**Figure 2.**
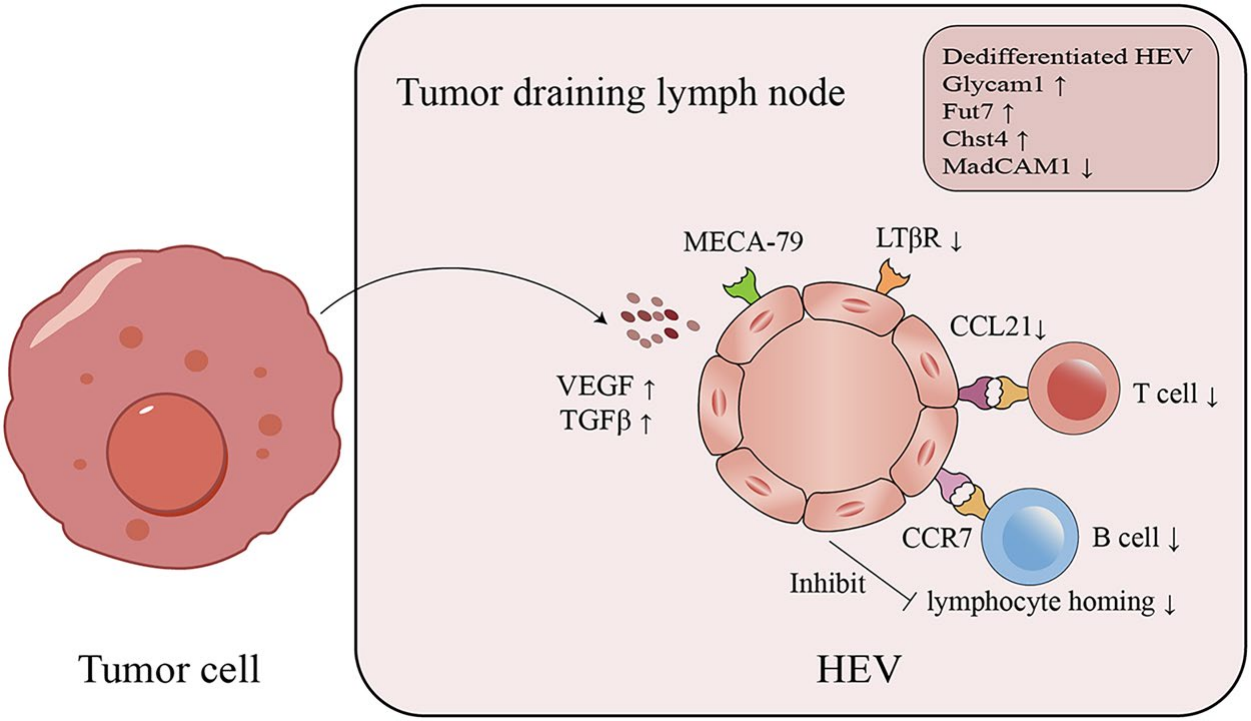
HEV in tumor draining lymph nodes.

Normal LNs consist of immune cells, fibroblastic reticular cells (FRCs), endothelial cells, and other components, connected to the outside through a lymphatic network that rapidly transmits tissue-derived signals (e.g. soluble antigens, lipids, metabolites, extracellular vesicles, and leukocytes) [37]. These components help maintain LN homeostasis and facilitate normal immune responses. Studies have shown that the TDLN microenvironment undergoes extensive remodeling before the arrival of tumor cells, involving changes in the vascular system, lymphatic system, and fibroblastic matrix [38]. As part of this microenvironment, HEVs in TDLNs are affected. Qian et al. confirmed that prior to tumor cell metastasis, the lumen diameter of HEVs increases, filled with red blood cells, the vessel wall becomes thinner, and MECA-79 expression is downregulated, indicating that HEVs gradually lose their columnar endothelial cell characteristics and transform into regular endothelial cells [39]. This dedifferentiation of HEVs leads to impaired lymphocyte homing function, further affecting the immune response in TDLNs and creating an immunosuppressive microenvironment favorable for tumor cell arrival [40] (Figure 2). LTβR expression downregulation may be a key molecule regulating HEV dedifferentiation. Studies have found that LTβR deficiency is a critical factor in inducing HEV dedifferentiation and further reduces the expression of CCL19 and CCL21, which are signals essential for HEV formation [13]. In addition, we found that tumor-derived VEGF-D can activate VEGFR2 and trigger HEV dedifferentiation, which in turn impairs lymphocyte homing and facilitates lymph node metastasis [41].

However, how HEVs undergo dedifferentiation in TDLNs and lead to impaired lymphocyte homing, resulting in reduced lymphocytes in the LNs, remains unclear. What specific mechanisms drive HEV dedifferentiation in TDLNs before tumor cells metastasize? These questions have not been fully addressed. Recently, researchers have proposed several mechanisms that might regulate HEV dedifferentiation: (1) Tumor antigens carried by DCs or inflammatory cytokines secreted by the tumor may lead to the expansion of TDLNs, further inducing the proliferation and separation of FRCs and the expansion of HEVs [42]. (2) Tumor-secreted factors, drained *via* lymphatic vessels to TDLNs, induce extensive remodeling of the LN microenvironment (including FRCs and HEVs), causing HEV dedifferentiation. The dedifferentiation of HEVs in TDLNs is also characterized by a reduction in the expression of CCL21 and CCL19 on HEV surfaces [43], which affects T-cell recruitment and leads to a decrease in lymphocytes within the LN [44].

Changes in the TDLN microenvironment occur even before tumor cells arrive. Studies have shown that injection of dead tumor cells into mice did not induce the formation of pre-metastatic niches in LNs [45]. However, when viable tumor cells were injected, significant microenvironment changes were observed in TDLNs, suggesting that tumor-secreted factors may play an important role in regulating the LN microenvironment [46]. Tumor-derived secretory factors help establish pre-metastatic niches in the LNs.

A clinical study has shown that HEV dedifferentiation in TDLNs, regardless of LN metastasis status, predicts poor prognosis for cancer patients [47]. This has been confirmed in patients with breast cancer, melanoma, and squamous cell carcinoma. Additionally, during LN metastasis, a decrease in the expression of BMP4 and DKK1 on the surface of HEVs has been observed, although their exact roles in HEVs remain to be clarified [48,49]. In conclusion, HEV dedifferentiation in TDLNs leads to impaired lymphocyte homing and is associated with the establishment of pre-metastatic niches in LNs. However, the role of TDLN HEVs in the dissemination of tumor cells is not yet well established and requires further investigation.

## HEVs in tumors

Recent research has shown that HEVs can appear within tumors and contribute to the formation of tertiary lymphoid structures (TLS). However, HEVs alone do not constitute TLS; rather, they are part of a broader immune microenvironment that includes various immune and stromal cells, which together create a functional TLS in these sites [50]. Generally, TLSs are organized aggregates of immune cells that form in non-lymphoid tissues under non-physiological conditions [51]. While the presence of TLSs has been associated with anti-tumor immune responses in some cancers, their role is not universally beneficial. For example, in renal cell carcinoma, the presence of TLSs correlates with a worse prognosis [52]. Moreover, the prognostic impact of TLSs is often determined by their maturation state and the specific immune cells present, rather than merely their presence. The presence of MECA-79^+^ HEVs alone is insufficient to define a functional TLS, as the full architectural and cellular composition of the structure is required for its immune activity [53]. However, in contrast to HEVs in LNs, TA-HEVs exhibit significant morphological variability. They can range from flat to cuboid shapes, with the latter tending to show a more mature phenotype, accompanied by more lymphocyte accumulation [54]. Some studies have used *in vivo* microscopy techniques to visualize TA-HEVs during tumor immunotherapy. During this period, lymphocytes were observed migrating through TA-HEVs into tumors to exert anti-tumor effects. Studies have shown that approximately 10% of lymphocytes migrate along non-HEV tumor blood vessels, while nearly 40% of lymphocytes roll along TA-HEVs, indicating that TA-HEVs are the primary conduit for transporting effector cells during anti-tumor treatments [7]. Furthermore, research has confirmed that TA-HEVs are significantly associated with the response to anti-tumor immunotherapy, suggesting that TA-HEVs may serve as a favorable prognostic factor in cancer patients.

## The potential therapeutic value of HEVs in tumors

HEVs, due to their unique ability to mediate lymphocyte trafficking, coupled with their specific marker, the MECA-79 antigen, hold significant therapeutic promise in the context of tumors [55]. As a target for lymphocyte-mediated cancer immunotherapies, HEVs could be leveraged in combination with immune checkpoint inhibitors, chimeric antigen receptor (CAR)-T cell therapy, various immune modulators, monoclonal antibodies, and experimental therapeutic vaccines [56]. In inflammatory diseases, preclinical models have demonstrated the therapeutic benefit of targeting HEV-like vessels. In an asthma sheep model, Rosen et al. found that intravenous injection of MECA-79 antibodies could prevent allergen-induced airway hyperresponsiveness and inhibit leukocyte aggregation in bronchoalveolar lavage fluid [57]. Additionally, in a mouse model of Sjögren’s syndrome, treatment with LTβR-Ig was associated with the inhibition of MECA-79^+^ HEV-like vessels and improved salivary and lacrimal gland function [58]. These studies indicate the significant application value of targeting HEVs in inflammatory diseases.

TA-HEVs are the primary route for lymphocyte entry into primary tumors, and thus, stimulating the maturation of TA-HEVs can enhance anti-tumor immune responses and improve clinical outcomes [59,60]. Immune checkpoint blockade therapies, using antibodies against PD-1 and CTLA-4, have been shown to facilitate the continuous recruitment of T cells into tumors, contributing to significant anti-tumor effects [61]. LTβR has been identified as a key target for inducing the maturation of TA-HEVs. Reports have shown that LTβR agonists induce TA-HEV formation and enhance lymphocyte infiltration in various mouse tumor models. In a glioblastoma model, the combined treatment of LTβR agonists, VEGFR2 inhibitors, and PD-L1 inhibitors produced a significant anti-tumor response [62]. Moreover, the use of STING agonists (ADU-S100) or PARP inhibitors (BMN 673) has been found to induce the generation of MECA-79^+^ TA-HEVs in mouse tumor models, promote lymphocyte recruitment, and improve the effectiveness of immunotherapies [63]. Dimberg developed an AAV-LIGHT approach that targets LIGHT, which helps activate HEVs within glioblastoma, promotes lymphocyte homing, and enhances antitumor effects [64]. Komatsu et al. simultaneously stimulated the expression of LTβR and STING, inducing the formation of TA-HEVs, markedly increasing the homing of intratumoral CD4^+^ T cells and memory CD8^+^ T cells, and enhancing antitumor efficacy [65]. Blanchard et al. recently developed Fc-optimized anti-CTLA-4 antibodies that remodel tumor vasculature and increase TA-HEVs, significantly enhancing the infiltration of CD4^+^ and CD8^+^ T cells [66]. Therefore, stimulating the proliferation of TA-HEVs to enhance anti-tumor immune responses can effectively improve tumor prognosis, making it an attractive strategy for cancer treatment.

A recent study found that in pancreatic ductal adenocarcinoma, utilizing the formation of HEVs with MECA-79-conjugated nanoparticles (MECA-79-NP) allowed for targeted delivery of chemotherapy drugs like paclitaxel directly to the tumor, resulting in effective anti-tumor effects [67]. This offers an alternative anti-tumor strategy, where the specific marker MECA-79 and the characteristic expression of HEVs within the tumor can be exploited to enhance the concentration of anti-cancer drugs at the tumor site, thereby improving anti-tumor efficacy and clinical outcomes. The use of nanoparticle-based drug delivery targeting HEVs is considered a promising approach for modulating immune responses in primary immune-mediated diseases, such as transplant rejection [68]. Specific targeting strategies for HEVs not only apply to the primary tumor but can also target HEVs in metastatic sites, including LNs and distant metastatic organs. A recent study developed a novel monoclonal antibody targeting PNAd (MHA112), and when conjugated with paclitaxel, it effectively delivered the drug to tumors, TDLNs, and metastatic lesions. This therapy significantly reduced primary tumor size and metastasis in breast cancer and pancreatic cancer mouse models [69]. Thus, HEV-targeted drug delivery platforms can simultaneously deliver anti-cancer drugs to both the primary tumor and metastatic sites, achieving dual inhibition of primary tumor growth and metastasis.

Studies have confirmed that HEVs in TDLNs undergo dedifferentiation, gradually losing their characteristic morphology and function, which prepares the environment for LN metastasis of tumor cells [39]. Stacker’s work demonstrated that the loss of BMP4 in TDLNs marks HEV dedifferentiation, manifesting as flattened and dilated vessels with altered proliferative responses [48]. However, whether BMP4-induced HEV dedifferentiation impairs lymphocyte trafficking and creates an immunosuppressive microenvironment favorable for tumor metastasis remains unclear. Therefore, based on the phenomenon of HEV dedifferentiation and lymphocyte trafficking in TDLNs, we hypothesize that reversing HEV dedifferentiation could improve the immune microenvironment in TDLNs, which may effectively prevent tumor metastasis. This could represent a critical strategy for future tumor metastasis prevention and treatment.

## Conclusion

In summary, HEVs, as a unique subset of the vascular system, play a crucial role in the process of immune cell trafficking due to their distinctive columnar endothelial cell morphology and lymphocyte homing function. The specific marker MECA-79, which is key to identifying HEVs, serves as an essential target for specific therapies. Furthermore, HEVs regulate lymphocyte homing and infiltration through specific molecular mechanisms, influencing immune responses within the tumor microenvironment, potentially serving as a basis for anti-tumor responses. This function of HEVs is not only critical in LN immune surveillance but also plays an important role in the tumor immune microenvironment. The formation of HEV-like vessels within tumors may enhance immune cell infiltration, contributing to the efficacy of tumor immunotherapy. On the other hand, the dedifferentiation of HEVs in TDLNs represents a valuable research direction, and reversing HEV dedifferentiation to improve the TDLN immune microenvironment could offer new strategies for preventing tumor metastasis. Therefore, investigating the mechanisms behind HEV formation and their role in tumors will provide new strategies and targets for tumor immunotherapy and LN metastasis management.

## Data Availability

Data sharing is not applicable to this article as no new data were created or analyzed in this study.
